# Human Umbilical Cord-Derived Mesenchymal Stem Cells Utilize Activin-A to Suppress Interferon-γ Production by Natural Killer Cells

**DOI:** 10.3389/fimmu.2014.00662

**Published:** 2014-12-29

**Authors:** Debanjana Chatterjee, Nicole Marquardt, Dejene Milkessa Tufa, Tim Hatlapatka, Ralf Hass, Cornelia Kasper, Constantin von Kaisenberg, Reinhold Ernst Schmidt, Roland Jacobs

**Affiliations:** ^1^Department of Clinical Immunology and Rheumatology, Hannover Medical School, Hannover, Germany; ^2^Institute of Technical Chemistry, Leibniz University of Hannover, Hannover, Germany; ^3^Laboratory of Biochemistry and Tumor Biology, Clinic of Obstetrics and Gynecology, Hannover Medical School, Hannover, Germany; ^4^Department of Biotechnology, University of Natural Resources and Life Science, Vienna, Austria; ^5^Department of Obstetrics, Gynecology and Reproductive Medicine, Hannover Medical School, Hannover, Germany

**Keywords:** UC-MSC, activin-A, suppression, NK cell, IFN-γ production, T-bet

## Abstract

Following allogeneic hematopoietic stem cell transplantation (HSCT), interferon (IFN)-γ levels in the recipient’s body can strongly influence the clinical outcome. Human umbilical cord-derived mesenchymal stem cells (UC-MSCs) are lucrative as biological tolerance-inducers in HSCT settings. Hence, we studied the molecular mechanism of how UC-MSCs influence natural killer (NK) cell-mediated IFN-γ production. Allogeneic NK cells were cultured in direct contact with UC-MSCs or cell-free supernatants from mesenchymal stem cell (MSC) cultures (MSC-conditioned media). We found that soluble factors secreted by UC-MSCs strongly suppressed interleukin (IL)-12/IL-18-induced IFN-γ production by NK cells by reducing phosphorylation of STAT4, NF-κB, as well as T-bet activity. UC-MSCs secreted considerable amounts of activin-A, which could suppress IFN-γ production by NK cells. Neutralization of activin-A in MSC-conditioned media significantly abrogated their suppressive abilities. Till date, multiple groups have reported that prostaglandin (PG)-E2 produced by MSCs can suppress NK cell functions. Indeed, we found that inhibition of PGE2 production by MSCs could also significantly restore IFN-γ production. However, the effects of activin-A and PGE2 were not cumulative. To the best of our knowledge, we are first to report the role of activin-A in MSC-mediated suppression of IFN-γ production by NK cells.

## Introduction

Interferon (IFN)-γ is a pleiotropic cytokine that modulates a diverse array of biological functions and is secreted by activated T cells and natural killer (NK) cells (1). The effect of IFN-γ on the outcome of hematopoietic stem cell transplantation (HSCT) is divaricated and controversial. Some reports suggest that IFN-γ can support graft vs. leukemia activity and prevent transplant rejection while others conclude that it can worsen graft vs. host disease (GvHD) by boosting recipient T cell proliferation [as reviewed in Ref. (2)]. In the early reconstitution phase following HSCT, NK cells form the major population of lymphocytes (3). A mouse model of allogeneic bone marrow transplantation (BMT) showed that NK cell-derived IFN-γ can contribute to a heightened anti-tumor activity by allografts (4). Thus, the ability of NK cells to secrete levels of IFN-γ might significantly affect the clinical outcome of a HSCT. Recently, interest is rising in using mesenchymal stem cells (MSCs) to prevent GvHD, and facilitate engraftment in HSCT due to their immunomodulatory nature. Co-transplanted immunosuppressive MSCs might influence the transplantation prognosis by modulating the levels of IFN-γ released by NK cells. The majority of studies reporting on mechanistic clues behind MSC-mediated inhibition of NK cells used bone marrow derived (BM)-MSCs. Although BM-MSCs are commonly used in clinical trials, umbilical cord-derived mesenchymal stem cells (UC-MSCs) can prove to be more suitable source of transplantable MSCs due to their high-proliferative potential, and ease of isolation (5). However, the molecular mechanism of how UC-MSCs affect the IFN-γ producing capacity of NK cells is unknown and is investigated in this study.

Mesenchymal stem cells isolated from bone marrow, tonsils, muscles, and dental pulps are known to produce activin-A (6). Activin-A is a transforming growth factor (TGF)-β superfamily member, which can suppress IFN-γ production possibly by reduction of T-bet levels in NK cells (7). We found that UC-MSCs also produce activin-A. Therefore, we investigated the contribution of activin-A in suppression of NK cells. Furthermore, prostaglandin (PG)-E2 produced by cyclooxygenase (COX)-2 activity in MSCs have been shown to be important in inhibition of NK cell cytotoxicity and proliferation (8, 9). In these studies, the mechanism of IFN-γ suppression was not outlined. A study on induced pluripotent stem (iPS) cell-derived MSCs reported that blocking of COX-2 failed to significantly restore interleukin (IL)-2-stimulated IFN-γ production by MSC-pretreated NK cells (10). The enzyme COX-1 is also able to release PGE2, and MSCs are known to express COX-1 (11). Noone et al. found that blocking COX-1 activity in BM-MSCs can largely restore IL-2/IL-15 stimulated IFN-γ production by NK cells (12). Therefore, we probed for the contribution of COX-1/COX-2 in UC-MSC-mediated suppression of IL-12/IL-18-induced IFN-γ production by NK cells.

## Materials and Methods

### Isolation and culture of UC-MSCs

This study was approved on 26th February, 2009 by the Institutional Review Board (Project No. 3037) in an extended permission # 443. With written consent, MSCs were isolated from human umbilical cords obtained from full-term infants (38–40 gestation weeks) by explant culture, expanded and cryopreserved as described elsewhere (13, 14). UC-MSCs were cultured in αMEM supplemented with 10% human serum and 50 μg/ml streptomycin, 50 U/ml penicillin, 1 mM glutamine, and 0.5 mM sodium pyruvate (Biochrom, Berlin, Germany). UC-MSCs were identified by high expression of CD73, CD90, and CD105 and lack of CD14, CD19, CD34, CD45, and HLA-DR (15). The ability of the UC-MSCs to undergo chondrogenic, adipogenic, and osteogenic differentiation was determined as described before (data not shown) (16). Hence, the MSCs were found to fulfill all the criteria recommended by the International Society for Cellular Therapy (17).

### Isolation of NK cells from peripheral blood mononuclear cells

Heparinized blood samples obtained from healthy consenting donors were diluted with an equal volume of phosphate buffered saline (PBS). Peripheral blood mononuclear cells (PBMCs) were separated by centrifugation over a ficoll-hypaque gradient and stained with anti-CD56 and CD3 antibodies. NK cells (CD56^+^CD3^−^ lymphocytes) were sorted using MoFlo Cell Sorter (Beckman Coulter).

### Cell culture

Umbilical cord-derived mesenchymal stem cells were seeded in 24- or 48-well plates and allowed to adhere for 24 h. Subsequently, freshly isolated allogeneic NK cells were added at a MSC:NK-ratio of 1:10 and co-cultured for 16 h. Cell-free supernatants from the NK-MSC co-cultures or MSC cultures without NK cells were frozen to analyze the role of soluble factors as MSC-NK-conditioned medium (NK-MSC cm) or MSC-conditioned media (MSC cm), respectively. NK cells exposed to MSCs or different conditioned media were collected, and tested for IFN-γ production. NK cells cultured alone in unconditioned media were used as controls. To induce IFN-γ production, NK cells were stimulated with IL-12 (10 ng/ml) or IL-18 (10 ng/ml) alone or in combination. Brefeldin A (Sigma) was added after 1 h, and the incubation was continued for 3 additional hours. IFN-γ production was analyzed by intracellular cytometric analysis. To neutralize activin-A, follistatin (R&D Systems) was added to MSC cm at a final concentration of 0.4 μg/ml. To block PGE2 production, indomethacin (R&D Systems) was added to MSC cultures at a concentration of 20 μM. Cell-free supernatants were generated from these cultures and are referred to as INDO-MSC cm. MSC cm was also collected from the same MSCs as controls.

### Detection of proteins using flow cytometry

The following anti-human mAbs have been used: CD3 PE (UCHT1) (Beckman Coulter), CD3 PerCP (SK7), CD11b PE (ICRF44), CD14 APC (M5E2), CD56 PE (MY31), IFN-γ FITC (B27), IL-12Rbeta1 PE (2.4E6), NFκB p65 PE (pS529), STAT4 PE (38/p-STAT4) (BD Biosciences), CD56 APC (HCD56), T-bet PE (4B10) (Biolegends), CD90 APC (5E10), IL-18Ralpha PE (H44) (eBiosciences), HLA-DR FITC (AB3) (Dako), CD44 FITC (HI44a), and CD105 FITC (MEM-226) (Immunotools). For detection of IFN-γ, after surface staining with anti-CD3 and CD56, NK cells were fixed with 4% paraformaldehyde (Merck) for 10 min at room temperature. The cells were perforated in 0.1% saponin buffer (PBS supplemented with 0.1% saponin (Riedel-de-Haën) and 0.01 M HEPES (Roth), and fluorochrome-tagged antibodies against IFN-γ or T-bet were added. After 30 min of incubation and three washes, cells were analyzed by flow cytometry. Each flow cytometric analysis was controlled with isotype-matched mAbs.

For detection of phosphorylated NF-κB p65 and STAT4 (pSTAT4), NK cells were stimulated with IL-12 and IL-18 for 15 min, washed and fixed immediately with 4% paraformaldehyde and BD Lyse/Fix Buffer 1×, respectively. pSTAT was detected using BD Phosflow anti-STAT4 antibody after permeabilizing the cells with BD Perm Buffer III, according to manufacturer’s protocol. Phosphorylated NF-κB p65 was detected with BD Phosflow anti-NF-κB p65 antibody after permeabilizing the cells with 0.1% saponin buffer as described previously. All flow cytometry-based experiments were performed on FACS Calibur using Cell-Quest Pro Software. Offline data analyses were done on Summit 5.1 software (Beckman Coulter).

### Activin-A enzyme-linked immunosorbent assay

Activin-A was measured in culture supernatants by sandwich enzyme-linked immunosorbent assay (ELISA) technique using a commercially available ELISA kit (R&D Systems), according to the manufacturer’s protocol. Concentrations were calculated by comparison with known activin-A standards using a free five parameter logistic curve fitting program^1^.

### T-bet DNA-binding ELISA

NK-92 cells were expanded in presence of 200 IU/ml of IL-2 followed by IL-2 deprivation for 2 days. The cells were cultured for additional 16 h, with or without MSC cm, and subsequently stimulated with IL-12 (10 ng/ml) and IL-18 (10 ng/ml) in fresh media. Nuclear proteins from NK-92 cells were extracted using a commercially available protein extraction kit (Nuclear Extract Kit, Active Motif) according to the manufacturer’s protocol. The amount of protein was quantitated using the Coomassie (Bradford) Protein Assay. The protein yield was between 0.1 and 0.15 mg at 2–3 mg/ml from 10^7^ NK-92 cells. The nuclear proteins were added to a 96-well plate coated with oligonucleotide containing T-bet consensus binding site (TransAM T-bet, Active Motif). At this step, the activated T-bet present in the nuclear extracts is expected to bind to the oligonucleotides coated on the plate. Bound T-bet was subsequently detected using anti T-bet ELISA, according to manufacturer’s protocol.

### Statistical analyses

We performed paired two-tailed *t*-tests or ANOVA with Bonferroni post-test (to compare more than two groups) using GRAPHPAD PRISM V5.00 Software. Levels of significance are shown as *p*-values (*p < 0.05, **p < 0.01, ***p < 0.001). Bar graphs represent mean ± SEM.

## Results

### MSC-induced suppression of IFN-γ production in NK cells

**Figure 1:**
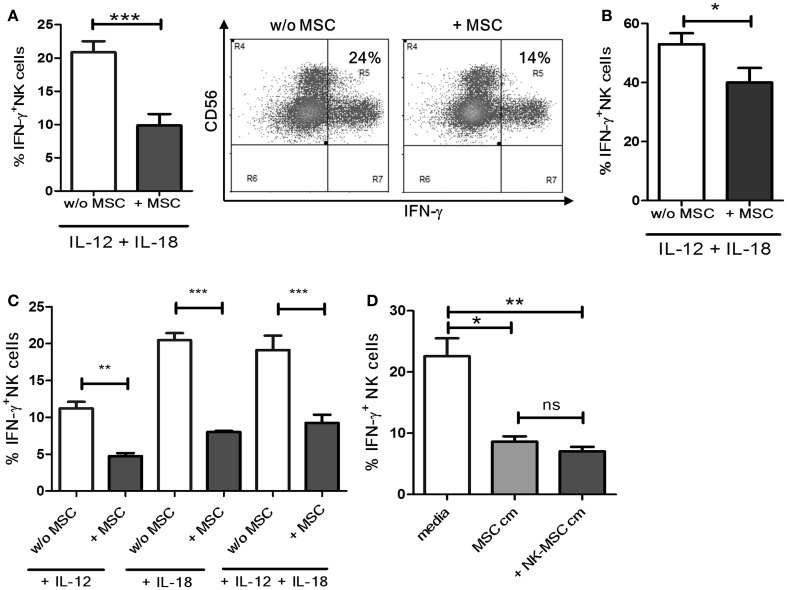
**Suppression of IFN-γ production by NK cells**. **(A)** NK cells cultured overnight with or without MSCs were stimulated with IL-12 and IL-18 in culture for 4 h. Brefeldin A was added after 1 h of culture. Cells were surface and intracellularly stained, followed by flow cytometry-based analysis of IFN-γ production (*n* = 6). The dot plot depicts IFN-γ^+^ NK cells from one representative experiment. **(B)** NK cells cultured overnight with or without MSCs were stimulated with IL-12 and IL-18 in culture for 18 h. The bar graphs represent the percentage of IFN-γ producing NK cells (*n* = 4). **(C)** NK cells were cultured with or without MSCs, and their IFN-γ producing ability in response to IL-12 alone, or IL-18 alone, or to the combination of IL-18 and IL-12 was measured (*n* = 4). **(D)** NK cells were cultured in MSC-conditioned media or NK-MSC-conditioned media, and their IFN-γ producing ability in response to IL-12 and IL-18 was measured (*n* = 4).

Natural killer cells were cultured with or without MSCs for 16 h and stimulated with IL-12 and IL-18 for 4 h. A significant reduction in the IFN-γ production was observed in NK cells pre-cultured with MSCs compared to NK cells cultured alone (Figure 1A, representative dot plots). Next, we investigated if the suppressive effect of MSCs is maintained under prolonged stimulation by IL-12 and IL-18. We activated NK cells, pre-cultured in the presence or absence of MSCs, with IL-12 and IL-18 for 18 h instead of 4. As shown in Figure 1B, NK cells pre-cultured with MSCs exhibited significant suppression even after prolonged stimulation. However, the suppression was not as potent as observed in the 4 h stimulation protocol. We further investigated if the MSCs caused the NK cells to be less responsive to stimulation by IL-12 or IL-18 or the combination of the two. We found that NK cells exposed to MSCs became significantly less responsive when used alone or in combination (Figure 1C). To ascertain the role of soluble factors in the observed immunosuppression, we cultured NK cells overnight in cell-free supernatants from MSC cultures and NK-MSC co-cultures. We found that both supernatants were similarly potent in suppressing IFN-γ production by NK cells (Figure 1D).

### MSC-mediated inhibition of IL-12 and IL-18 receptor signaling in NK cells

**Figure 2:**
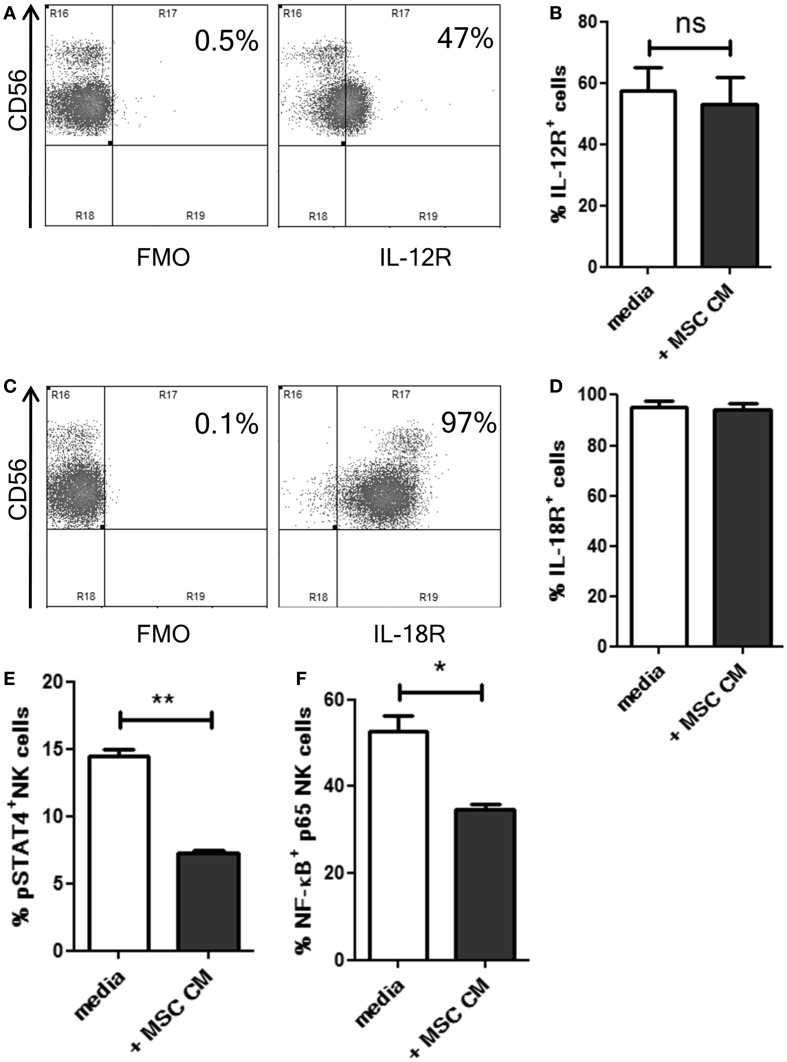
**Effect of UC-MSCs on IL-12 and IL-18 receptor signaling in NK cells**. **(A–D)** NK cells were cultured in normal media or MSC cm. NK cells were washed, IL-12R and IL-18R expression was analyzed. The dot plots depict the levels of IL-12R **(A)** and IL-18R **(C)** on normal NK cells. The bar graphs represent the percentage of IL-12R^+^ [**(B)**; *n* = 5] and IL18R^+^ [**(D)**; *n* = 5] NK cells cultured with or without MSC cm. **(E,F)** NK cells were cultured in normal media or MSC cm, followed by stimulation with IL-12 and IL-18 to induce phosphorylation events downstream of IL-12R and IL-18R activation (*n* = 3). Intracellular levels of pSTAT **(E)** and activated NF-κB **(F)** was measured in the NK cells.

Our results suggested that MSCs inhibited the responsiveness of NK cells to IL-12 and IL-18 using some soluble factors. Hence, we examined if MSC secreted factors affected the expression of IL-12 and IL-18 receptors (IL-12R and IL-18R, respectively) on the NK cells. Following overnight culture in MSC cm or normal media, the expression of IL-12R beta1 and IL-18R alpha was analyzed on NK cells. CD56^dim^ NK cells showed higher expression of IL-12R compared to CD56^bright^ cells (Figure 2A). MSC cm was found to have no effect on IL-12R expression levels on NK cells (Figure 2B). Almost all NK cells stained positive for IL-18R (Figure 2C) and treatment with MSC cm did not alter IL-18R expression on NK cells (Figure 2D). Next, we examined if MSC cm affected the downstream signaling of these receptors without affecting their surface expression. Phosphorylation of STAT4 and NF-κB p65 are commonly reported signaling events associated with the activation IL-12R and IL-18R, respectively (18–20). Following overnight culture in MSC cm or normal media, we stimulated the NK cells with IL-12 and IL-18 for 15 min. Upon analyzing the intracellular levels of pSTAT4 and activated NF-κB in the NK cells, we found that MSC cm could significantly abrogate both IL-12R and IL-18R downstream signaling. Pre-treatment with MSC cm significantly reduced pSTAT4 and activated NF-κB content of IL-12 and IL-18 stimulated NK cells (Figures 2E,F; Figure S2 in Supplementary Material).

### Co-culture with UC-MSC induces downregulation of T-bet in NK cells

**Figure 3:**
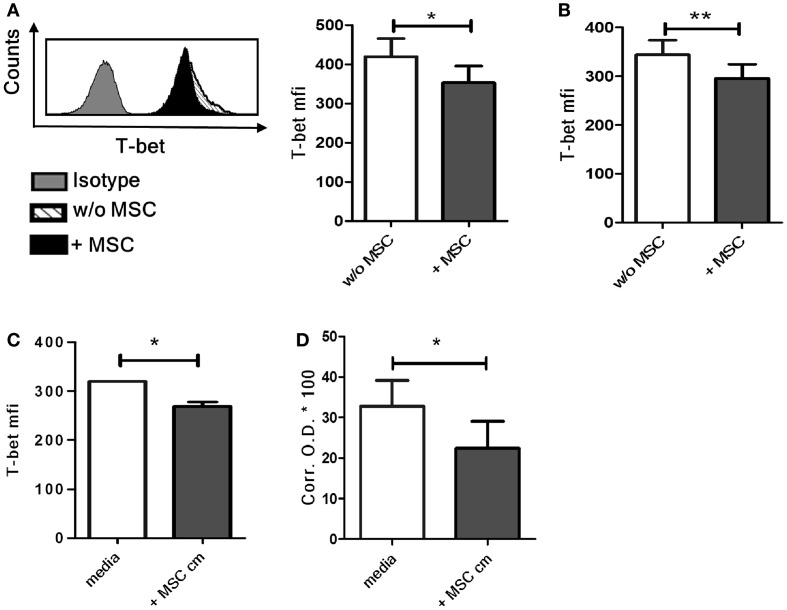
**Effect of UC-MSCs on T-bet activity in NK cells**. **(A)** NK cells cultured with or without MSCs were harvested and stained for intracellular T-bet (*n* = 4). **(B)** NK cells cultured with or without MSCs were harvested, stimulated with IL-12 and IL-18 for 4 h and stained for intracellular T-bet (*n* = 5) **(C)** NK cells were cultured in MSC-conditioned media (MSC cm) or normal media for 16 h and stained for intracellular T-bet (*n* = 3). **(D)** NK-92 cells were expanded in presence of IL-2 and subsequently cultured without IL-2 for 2 days. The NK-92 cells were collected, washed, and cultured for 16 h with MSC cm or without MSC cm. The nuclear proteins from NK-92 cells were extracted, quantitated, and frozen. The amount of activated T-bet present was detected using T-bet DNA-binding ELISA with a standard colorimetric read-out. The graph represents the corrected OD 450 values of each sample multiplied by a factor of 100 (*n* = 4).

The transcription factor T-bet is the master regulator of IFN-γ production in T cells and NK cells (21). We found a significant decrease of basal T-bet levels in NK cells following co-culture with MSC compared to control NK cells cultured without MSCs (Figure 3A). When the NK cells were removed from the MSCs after overnight co-culture and then stimulated with IL-12 and IL-18, the difference in T-bet levels in NK cells cultured with or without MSCs was still maintained (Figure 3B). Similar decrease in T-bet levels was also seen in NK cells cultured with MSC cm (Figure 3C). We further examined if the exposure to MSC cm lowers the DNA-binding activity of T-bet in NK cells by nuclear protein isolation (containing activated T-bet) followed by T-bet DNA-binding ELISA. Since it is difficult to obtain large number of NK cells from peripheral blood, we used NK-92 cells instead of primary NK cells. Our experiments had so far focused on NK cell-MSC interaction in absence of any stimulatory cytokine while the NK-92 cells are normally expanded in presence of IL-2. These cells were therefore deprived of IL-2 for 2 days and then cultured for 16 h with or without MSC cm. The cells were then stimulated with IL-12 and IL-18 for 4 h. We first examined if this protocol resulted in a decrease of IFN-γ production NK-92 cells cultured in MSC cm. Indeed, the IL-2 deprived NK-92 cells are susceptible to the suppressive effect of MSC cm. MSC cm-treated NK-92 cells produced considerably less IFN-γ upon IL-12/IL-18 stimulation compared to NK-92 cells cultured in normal media (Figure S1 in Supplementary Material). Next, we proceeded to test for changes in T-bet DNA-binding activity. The IL-2 deprived NK-92 cells were cultured for 16 h with or without MSC cm. This was followed by stimulation with IL-12 and IL-18 for 4 h to activate T-bet and allow its nuclear translocation. Nuclear proteins were extracted from NK-92 cells and the extent of T-bet activation was assayed using DNA-binding ELISA. We observed that pre-treatment of NK-92 cells with MSC cm significantly reduced the T-bet activation levels compared to cells cultured without MSC cm (Figure 3D).

### Mechanism of IFN-γ suppression

**Figure 4:**
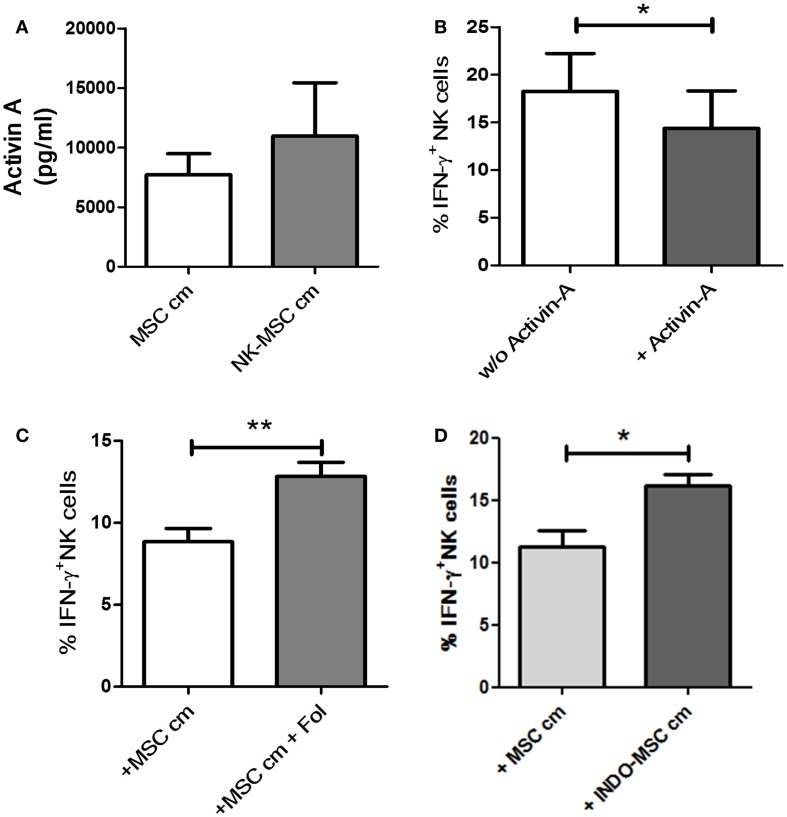
**Role of activin-A and PGE2**. **(A)** Using a sandwich ELISA, the amount of activin-A secreted by MSCs when cultured alone (MSC cm) or in presence of NK cells (NK-MSC cm) was evaluated (*n* = 4). **(B)** NK cells were stimulated with IL-12 and IL-18 in presence or absence of 50 ng/ml activin-A. Brefeldin A was added after 1 h of culture. Following 4 h of stimulation, the cells were stained for intracellular IFN-γ expression and analyzed by flow cytometry for IFN-γ production (*n* = 4). **(C)** NK cells were cultured in MSC cm in presence or absence of follistatin (Fol). NK cells were stimulated with IL-12 and IL-18 for 4 h and stained for intracellular IFN-γ (*n* = 7). **(D)** NK cells were cultured in MSC-conditioned media (MSC cm) or MSC cm generated in presence of indomethacin (INDO-MSC cm). The NK cells were stimulated with IL-12 and IL-18 for 4 h and stained for intracellular IFN-γ (*n* = 4).

Activin-A has been described to suppress IFN-γ production, without affecting NK cells’ perforin or granzyme content, possibly by reduction of T-bet levels in NK cells (7). MSC cm or NK-MSC cm was found to have no effect on the perforin or granzyme contents of NK cells (data not shown). Thus, we hypothesized that activin-A could be one of the molecules secreted by UC-MSC, which is critical for the observed inhibition of IFN-γ production by NK cells. Using a sandwich ELISA, we could demonstrate that UC-MSCs secreted activin-A constitutively. Upon exposure to NK cells, there was an increase in the release of activin-A. However, this increase did not reach significance (Figure 4A). Addition of activin-A to NK cell cultures could significantly reduce their IFN-γ producing ability in response IL-12/IL-18 (Figure 4B). Follistatin is an activin-binding protein that can neutralize the bioactivity of the latter (22). We added follistatin in MSC cm to neutralize activin-A. NK cells cultured in activin-A-neutralized MSC cm were significantly better at IFN-γ production compared to NK cells cultured in MSC cm (Figure 4C). This indicates that activin-A neutralization can abrogate the suppressive potential of MSC cm. We also blocked COX-2 activity in NK cell-MSC co-cultures using a pharmacological inhibitor (NS-398). COX-2 inhibition could not abolish MSC-induced suppression of IFN-γ production (Figure S3 in Supplementary Material). Recently, Noone et al. found that pharmacological blocking of COX-1 could alleviate BM-MSC-induced suppression of IFN-γ production by NK cells. To verify the role of COX-1 in UC-MSC-mediated suppression, we generated conditioned media from UC-MSC cultures in presence of COX-1 blocker, indomethacin (INDO-MSC cm). MSC cm was collected as control. Fresh NK cells were cultured in MSC cm or INDO-MSC cm for 16 h. The NK cells were then washed and stimulated with IL-12 and IL-18 for 4 h to induce IFN-γ production. Blocking PGE2 release using indomethacin could significantly restore NK cell IFN-γ production (Figure 4D). However, simultaneous blocking of activin-A and PGE2 failed to show additive effects (Figure S4 in Supplementary Material).

## Discussion

The inclination toward using MSCs for cell therapy is on the rise, and BM-MSCs have been shown to be useful for the treatment and prevention of GvHD, as well as facilitation of engraftment following HSCT in several clinical trials (23). The anti-inflammatory effect of MSCs, that helps control GvHD, is possibly mediated by the suppression of T cell proliferation (24). Following HSCT, the first lymphocyte population to reconstitute are NK cells and they can eliminate recipient leukemic cells which leads to significantly reduced rates of leukemia relapse (25) without causing GvHD. MSCs co-administered with HSC to suppress T cells are also likely to suppress NK cell functions, thereby eliminating their beneficial effects. Detailed understanding of MSC-immune system interaction is a prerequisite to efficient use of MSCs as biological immunosuppressant.

We found that NK cells pre-cultured with UC-MSCs exhibit significant deficit in their ability to secrete IFN-γ upon stimulation with IL-12 and IL-18 compared to NK cells cultured without MSCs. We observed that MSC cm could effectively suppress IFN-γ production. This indicated that some soluble factor, which dampens the IFN-γ synthesizing machinery, is secreted by the MSCs in large amounts even before coming in contact with NK cells. However, these factors did not affect IL-12 and IL-18 receptor expression on NK cells. Instead, MSC cm significantly reduced phosphorylation of signaling intermediates downstream of IL-12 and IL-18 receptor ligation by corresponding ligands. Previous exposure to MSC cm affected phosphorylation of STAT4 and NF-κB. We found significant decrease of basal T-bet levels in NK cells after being co-cultured in direct contact with MSC or MSC cm compared to control NK cells cultured in normal media. This reduction in T-bet was maintained in these NK cells even after stimulation with IL-12 and IL-18. The biological activity of a transcription factor largely depends on its ability to bind to DNA in order to activate transcription. Therefore, it was important to know if soluble factors from MSCs could regulate the DNA-binding ability of T-bet in NK cells. IL-2 deprived NK-92 cells were used instead of NK cells to study the DNA-binding activity of T-bet. We found that pre-exposure to MSC cm considerably reduced the ability of NK-92 cells to activate T-bet when subsequently exposed to IL-12 and IL-18. T-bet is one of the most important regulators of IFN-γ promoter activity. Hence, the observed defect in T-bet activation could possibly explain the significant reduction in IFN-γ production by NK cells when exposed to MSC derived soluble suppressants.

Activin-A has been described to suppress IFN-γ production by NK cells along with a decrease in T-bet levels without affecting NK cell cytotoxicity (7). UC-MSCs were found to produce considerable amounts of activin-A. Treatment of NK cells with activin-A inhibited their IFN-γ producing potential. Blocking activin-A in MSC cm using follistatin abrogated MSC cm-mediated suppression, and produced significant increase in percentage of IFN-γ producing NK cells. In our experimental settings, blocking PGE2 release using the selective COX-2 blocker had failed to restore IFN-γ production by NK cells. However, indomethacin mediated blocking could successfully alleviate MSC-induced suppression of IFN-γ production by NK cells. Indomethacin is a universal COX inhibitor but is relatively selective for COX-1. These results imply that COX-1-derived PGE2 is sufficient to suppress NK cell IFN-γ production. This explains why the blocking of PGE2 production by COX-2 alone (using NS-398) was ineffective in our culture conditions. This can also possibly explain why Giuliani et al. were unsuccessful in abrogating PGE2 mediated suppression using NS-398 (10). Our hypothesis seeks to resolve the controversy about the involvement of PGE2 in suppression of IFN-γ production. Our observations, thus, identify activin-A as yet another “partner in crime” in the growing list of strategies used by MSCs to suppress our immune system. These results indicate that UC-MSCs utilize activin-A as well as PGE2 (derived mainly from COX-1 activity) to reduce IFN-γ production by NK cells.

Although UC is an excellent source of MSCs (26) and several clinical trials using UC-MSCs are underway^2^, BM-MSCs are still the gold-standard for cell therapy (13, 27). Activin-A has been shown to be released by BM-MSCs as well as MSCs derived from tonsils, muscles, and dental pulps (6). Therefore, our findings might extend to the immunosuppression mediated by other MSC populations, including clinically relevant BM-MSCs. However, this remains to be investigated. Understanding the commonalities in the immunosuppression exerted by different MSC populations can help us stream-line our efforts toward improved MSC-based cell-therapies in future.

## Author Contributions

Contribution: Debanjana Chatterjee, Nicole Marquardt, Dejene Milkessa Tufa, Tim Hatlapatka, Ralf Hass, and Roland Jacobs performed experiments; Constantin von Kaisenberg contributed the umbilical cord, Tim Hatlapatka and Cornelia Kasper contributed vital reagents and discussed the data, Debanjana Chatterjee and Roland Jacobs analyzed the results and made the figures; Debanjana Chatterjee, Nicole Marquardt, Cornelia Kasper, Reinhold Ernst Schmidt, and Roland Jacobs designed the research; Debanjana Chatterjee wrote the paper.

## Conflict of Interest Statement

The authors declare that the research was conducted in the absence of any commercial or financial relationships that could be construed as a potential conflict of interest.

## Supplementary Material

The Supplementary Material for this article can be found online at http://www.frontiersin.org/Journal/10.3389/fimmu.2014.00662/abstract

## Footnotes

^1^http://www.readerfit.com

^2^http://clinicaltrials.gov
