# Supplementary material for: Human Umbilical Cord-Derived Mesenchymal Stem Cells Utilize Activin-A to Suppress Interferon-γ Production by Natural Killer Cells
Source: Front Immunol. 2014 Dec 29;5:662. doi: 10.3389/fimmu.2014.00662 (PMC4278046; doi:10.3389/fimmu.2014.00662)
Supplement: Supplementary file 1 [file Presentation_1.PDF]

Supplementary Material

Human umbilical cord-derived mesenchymal stem cells utilise activin-A to suppress Interferon-γ production by natural killer cells

Debanjana Chatterjee[1], Nicole Marquardt[1], Dejene Milkessa Tufa[1], Tim Hatlapatka[2], Ralf Hass[3], Cornelia Kasper [2,4], Constantin von Kaisenberg [5], Reinhold Ernst Schmidt[1] and Roland Jacobs[1 *]

[1]Dept. of Clinical Immunology and Rheumatology, Hannover Medical School, Hannover, Germany; [2]Institute of Technical Chemistry, Leibniz University of Hannover, Hannover, Germany; [3]Laboratory of Biochemistry and Tumor Biology, Clinic of Obstetrics and Gynecology, Hannover Medical School, Hannover, Germany; [4]University of Natural Resources and Life Science, Dept. of Biotechnology, Vienna, Austria; [5]Dept. of Obstetrics, Gynecology and Reproductive Medicine, Hannover Medical School, Hannover, Germany

[*]**Correspondence:**
R. Jacobs
Dept. of Clinical Immunology and Rheumatology,
Hannover Medical School,
Carl-Neuberg-Str. 1, 30625
Hannover, Germany.
Phone: +49-(0)511-532 3581, fax: +49-511-532 5648,
email: jacobs.roland@mh-hannover.de

1. Supplementary Figures

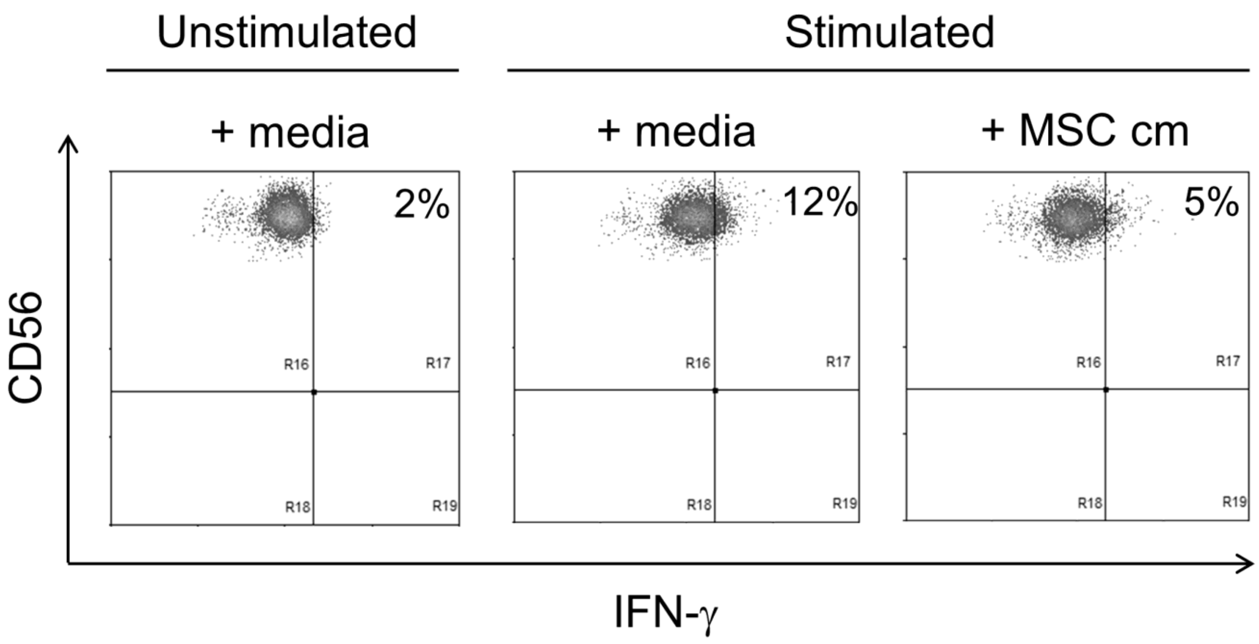

Figure S1 Effect of UC-MSCs on IFN-γ production in NK-92 cells. NK-92 cells were first expanded in presence of IL-2 and then cultured without IL-2 for two days. The cells were collected, washed and cultured for 16 hours with two different MSC cm or without MSC cm. These cells were again washed and cultured in fresh media in presence of IL-12 and IL-18 stimulation. Brefeldin A was added after 1 hour and the incubation was continued for 3 additional hours. Finally, the NK-92 cells were surface and intracellularly stained, followed by flow cytometry-based analysis of IFN-γ production. Representative dot plots depicting IFN-γ production by NK-92 cells are shown (n = 2)

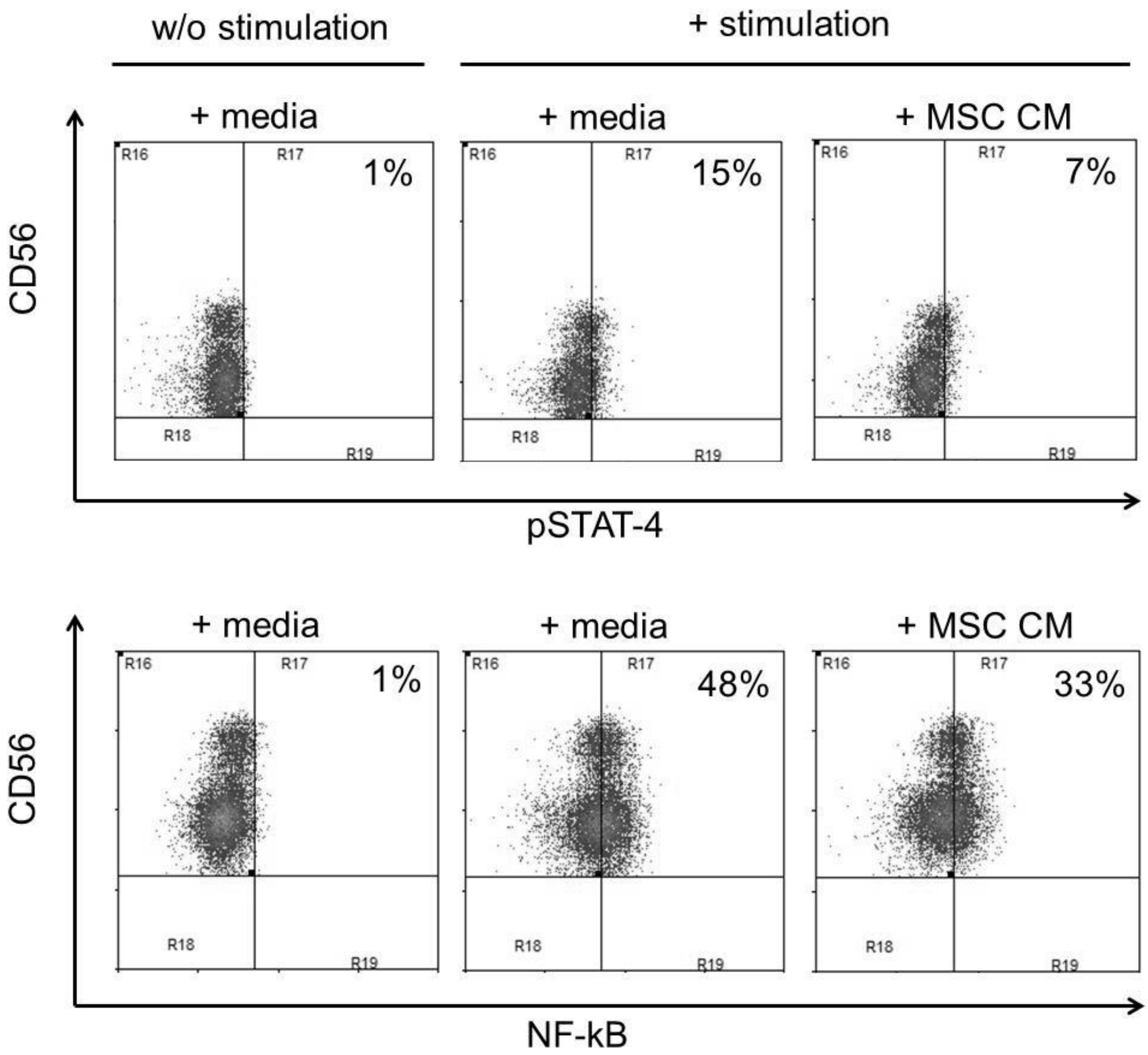

Figure S2 Effect of MSC cm on STAT4 and NF-κB activation in NK cells. NK cells were cultured in normal media or MSC cm, followed by stimulation with IL-12 and IL-18 to induce phosphorylation events downstream of IL-12R and IL-18R activation (n = 3). Intracellular levels of pSTAT-4 and activated NF-κB in were measured in the NK cells. Representative dot plots depicting the activation of STAT-4 and NF-κB induction are shown.

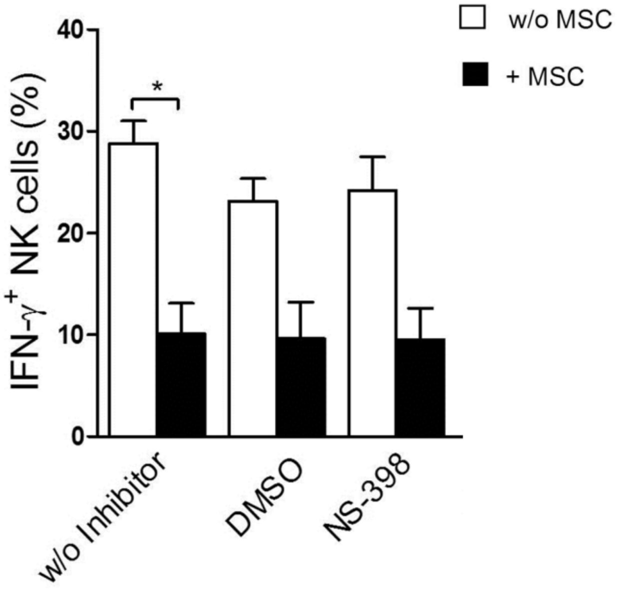

Figure S3 Effect of blocking COX-2 on UC-MSC-mediated suppression of IFN-γ production. NK cells were cultured with or without MSCs or co-cultured in presence of NS-398 or DMSO (vehicle control) followed by stimulation with IL-12 and IL-18 for 4 hours. Brefeldin A was added after 1 hour of culture. Cells were surface and intracellularly stained, followed by flow cytometry-based analysis of IFN-γ production.

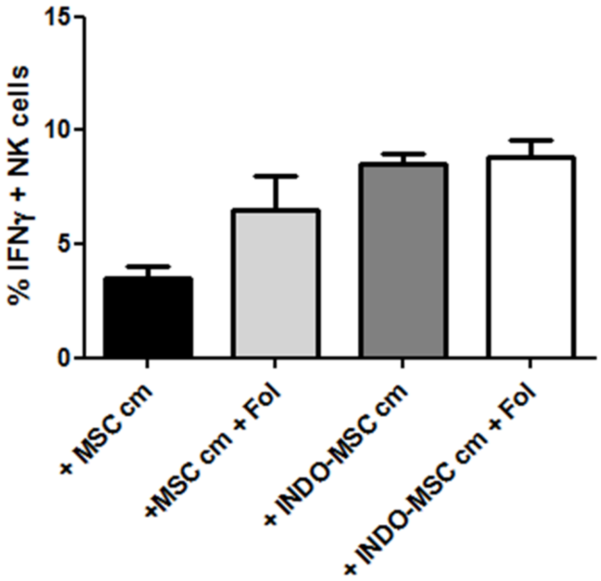

Figure S4 Simultaneous blocking of Activin-A and PGE-2. NK cells were cultured in MSC-cm or INDO-MSC cm in presence or absence of follistatin (Fol). NK cells were stimulated with IL-12 and IL-18 for 4 hours and stained for intracellular IFN-γ (n = 4)
